# p16^INK4A^ Expression in Condyloma Acuminata Lesions Associated with High-Risk Human Papillomavirus Infection

**DOI:** 10.31557/APJCP.2021.22.10.3219

**Published:** 2021-10

**Authors:** Gondo Mastutik, Alphania Rahniayu, Afria Arista, Dwi Murtiastutik, Nila Kurniasari, Trisniartami Setyaningrum, Anny Setijo Rahaju, Erna Sulistyani

**Affiliations:** 1 *Department of Anatomic Pathology, Faculty of Medicine, Universitas Airlangga, Surabaya, Indonesia. *; 2 *Department of Dermatology and Venereology, Faculty of Medicine, Universitas Airlangga, Surabaya, Indonesia.*; 3 *Department of Oral Medicine, Faculty Dentistry, Jember University, Jember, Indonesia *

**Keywords:** p16^INK4A^, high-risk HPV, low-risk HPV, condyloma, sexually transmitted diseases

## Abstract

**Objective:**

The objective of this study was to discover the possible correlation between p16INK4A expression and the LR/HR-HPV infection in condyloma acuminate (CA) lesions.

**Materials and Method:**

This cross-sectional study was conducted during January-December 2017 on 33 CA patients. The expression of p16^INK4A^ was detected by immunohistochemistry (IHC) staining. The positive interpretation was carried out by scoring which score 0 was negative, score 1 was sporadic, score 2 was focal, and score 3 was diffuses. The HPV genotypes were identified by reverse line blot, and 40 genotypes of HPV detected, including HR-HPV (HPVs 16, 18, 26, 31, 33,35, 39, 45, 51, 52, 53, 56, 58, 59, 66, 67, 68a, 68b, 69, 73, and 82) and LR-HPV (HPVs 6, 11, 40, 42, 43, 44, 54, 55, 61, 62, 64, 70, 71, 72, 81, 83, 84, 87, 89, and 90).

**Results:**

The expression of p16^INK4A^ was significantly correlated with HR-HPV infection. Patients infected with HR-HPV had 0.644 times higher possibility to express p16^INK4A^ gene compared to those infected with LR-HPV. LR-HPV genotypes detected in CA patients were HPVs 6, 11, 42, 61, 54, 81, 87, 89, and 90 and HR-HPV genotypes were HPVs 18, 26, 45, 51, 52, 67, 68B, 69, and 82. LR-HPV was found in 19/33 of patients and HR-HPV was in 14/33 of patients. The expression of p16^INK4A^ in CA lesions was diffuse in15.2% of patients, was focal in 24.2% of patients , was sporadic in 39.4% of patients were, and was negative in 21.2% of patients . In LR-HPV group, there was no diffuse expression, focal expression was observed in 15.8%, sporadic in 47.4%, and negative in 36.8%, while in HR-HPV group, p16^INK4A^ expression was detected in all lesions , in a way that its expression was diffuse in 35.7%, focal in 35.7%, and sporadic in 28.6%.

**Conclusion:**

IHC is a routine method in histopathological diagnosis, therefore the detection of p16^INK4A^ expression by IHC can be used as a biomarker for HR-HPV infection diagnosis.

## Introduction

Condyloma acuminata (CA) is the most frequent sexually transmitted disease worldwide (Santegoets et al., 2012). It is also known as genital warts or anogenital warts. These lesions are usually single or multiple appearing in the anogenital region and causing symptoms of itching, vaginal discharge, and bleeding. CA can be flat or lobulated and appears as pearl-like, filiform, plaque eruption, or cauliflower projection (Patel et al., 2013; Léonard et al., 2014). Its prevalence is increasing worldwide. The prevalence of CA in Italian female population during 2009-2010 was 3.8 cases per 1,000 women per year (Suligoi et al., 2017), in the UK population during 2010-2012 was 3.8% in males and 4.6% in females (Sonnenberg et al., 2019), and in the US population during 2013-2014 was 2.9% (Daugherty et al., 2018). A systematic review on the incident of anogenital warts showed that their prevalence ranged from 160 to 289 per 100,000 persons. New incident of anogenital warts ranged from 103 to 168 per 100,000 persons among males and 76 to 191 per 100,000 persons among females (Patel et al., 2013).

The most common cause of CA is Human Papillomavirus (HPV). There are two groups of HPV, including high risk (HR) HPV and low risk (LR) HPV. HR-HPV are HPV genotypes of 16, 18, 26, 31, 33, 35, 39, 45, 51, 52, 53, 56, 58, 59, 61, 73, and 82, and LR-HPV are HPV genotypes of 6, 11, 40, 42, 43, 44, 54, 61, 70, 72, and 81 (Braaten et al., 2008; Gutiérrez-Xicoténcatl et al., 2009). LR-HPV causes CA, but a study in China showed that the infection of HR-HPV was also found in CA lesions. In some cases, CA is caused by the combination of LR and HR-HPV infections (Lu et al., 2014). Persistent infection of LR or HR-HPV is a risk factor for the transformation of epithelial to benign hyperplasia or premalignant lesions. HR-HPV is related to the occurrence of malignancy in women, as cervical cancer (Santegoets et al., 2012). Some literature considers HR-HPV as the main causative agent responsible for the cervical cancer (Braaten et al., 2008). The detection of HPV genotype is very important to prevent, establish early diagnosis, and initiate the treatment in cervical cancer. Determination of LR or HR-HPV in anogenital warts can be used as a factor to predict the progression of lesions to benign or malignant lesions.

The E7 HPV protein plays a role in the cell transformation process. It binds to important proteins, such as the pRB and cyclin A/CDK2 complex, inhibiting the interaction between Rb and E2F. The E7 protein of HR-HPV deactivates pRB, resulting in the accumulation of p16^INK4A^ protein. The expression of p16^INK4A^ can also be considered as a marker of E7 gene activity (Izadi-Mood al., 2012; Romagosa et al., 2011). p16^INK4A^ plays a role in cell cycle regulation and it is involved in the processes of apoptosis, angiogenesis, cell invasion. This activity may be associated with overexpression in cancer (Romagosa et al., 2011). The expression of p16^INK4A^ is a marker to determine the prognosis of a malignancy caused by HPV infection (Missaoui et al., 2010), suggesting that p16^INK4A^ can be a specific marker for HPV infection and may correlate with the type of HR-HPV or LR-HPV. The objective of this study was to analyze the correlation between p16^INK4A^ expression and the LR-HPV or HR-HPV in CA lesions. This study identified the genotype of HPV and performed immunohistochemical analysis of p16^INK4A^ staining of. 

## Materials and Methods

*The samples collection*

This cross-sectional study was conducted at Outpatient Clinic of Department Dermatology and Venereology, Dr. Soetomo General Academic Hospital, Surabaya, Indonesia, from January 2017 to December 2017. The study was ethically approved by the Medical Ethic Research at Dr. Soetomo General Academic Hospital, Surabaya (ethical code: 382/Panke.KKE/V/2016).

All patients with CA, both male and female, who were willing to participate in the study, were included. Informed consent was obtained from the participants. Menstruating and pregnant women, those who suffered from an active pelvis and / or acute cervicitis, men or women with a diagnosis of HIV and AIDS, and those who were not willing to participate in the study were excluded.

The specimens were 33 tissues of CA. Tissue from each patient was divided into 2 parts, one part for tissue processing into paraffin block preparations followed by histopathological diagnosis and immunohistochemistry (IHC) staining, while another for the examination of the HPV genotype. Histopathological diagnosis of CA and analysis of IHC staining was performed by a pathologist. 

*Expression of p16* ^INK4A^

The expression of p16INK4A was detected by IHC staining using Anti-CDKN2A/ p16^INK4A^ Antibody (clone 1E12E10) IHC-plus™ LS-B5261 (LS Bio). The interpretation was positive if cells were stained in the nucleus or combined in the nucleus and cytoplasm. The assessment was carried out based on guidelines presented by Klaes et al. (2001) as follows:

Score 0 (negative): if cells were stained positive <1% of all cells

Score 1 (sporadic): if cells were stained positive <5% of all cells

Score 2 (focal): if cells wee stained positive <25% of all cells

Score 3 (diffuses): if cells were stained positive > 25% of all cells. 

*Genotyping of HPV*

Virus extraction was carried out from CA tissues using the QIAamp DNA Mini Kit (Qiagen) kit and according to the manufacturer’s protocol. Genotyping of HPV was performed by Polymerase Chain Reaction (PCR), then followed by reverse line blot using the Ampliquality HPV type express v 3.0 kit (Ab Analitica). This kit could detect 40 genotypes of HPV, including HR-HPV were HPV 16, 18, 26, 31, 33,35, 39, 45, 51, 52, 53, 56, 58, 59, 66, 67, 68 (68a, 68b), 69, 73, and 82 and LR-HPV were HPV 6, 11, 40, 42, 43, 44, 54, 55, 61, 62, 64, 70, 71, 72, 81, 83, 84, 87, 89, and 90.

*Statistical analysis*

The difference in the expression of p16^INK4A^ on CA patients with HR-HPV or LR-HPV groups infection was analyzed by Mann Whitney test (significant if p <0.05). The correlation between variables was analyzed by two-tailed Spearman’s rho (significant if p <0.05). 

## Results

*Characteristics of patient*

This study was performed on 33 patients suffering from CA. Patients’ characteristic, such as sex, age, sexual partner, duration of the symptoms, medical history, presence of lesion in the partner, history of treatment, and shape, type, and location of the lesion were investigated. The patients consisted of 12 (36.4%) males and 21 (63.6%) females. The patients aged from 18 to 64 years. The highest frequency was allocated to the age group of 15-24 years old (51.5%). Regarding sexual partner, it was found that 30/33 of the patients (90.9%) were heterosexual (men having sex with women), 2/33 (6.1%) were bisexual (having sex with the same and different sex), and 1/33 (3.0%) were homosexual (having sex with the same sex). Regarding symptoms duration, the highest frequency was allocated to the duration of 1to 3 months. Mostly, the patients experienced CA for the first time, there was no lesion in their partner, and received TCA therapy, respectively. Considering the shape, type, and location of the lesion, it was revealed that the highest frequencis were allocated to papule shape, multiple type of lesions, and gland penis for male and labia majora for female (Table1). 

*HPV genotype in CA lesions*

The genotype of HPV included the infection of HPV-HR and LR, both single and multiple infections. The single or multiple infections of HPV-LR was assumed as infection of LR- HPV. The multiple infection of LR-HPV with HR-HPV was assumed as infection of HR-HPV. The genotype of LR-HPV were HPV 6, 11, 42, 61, 54, 81, 87, 89, and 90, and of HR-HPV were 18, 26, 45, 51, 52, 67, 68B, 69, and 82. LR-HPV was found in 19/33 (57.6%) of the patients and HR-HPV was seen in 14/33 (42.4%) of the patients (Table 2). The most dominant HPV was HPV 11 that infected 24/56 (42.9%) times more, then followed by HPV 6 (16.1%) and HPV 18, HPV 51, and HPV 82 for 5.4%, respectively. 

*p16* ^INK4A^ *expression in CA lesions*

**Figure 1:**
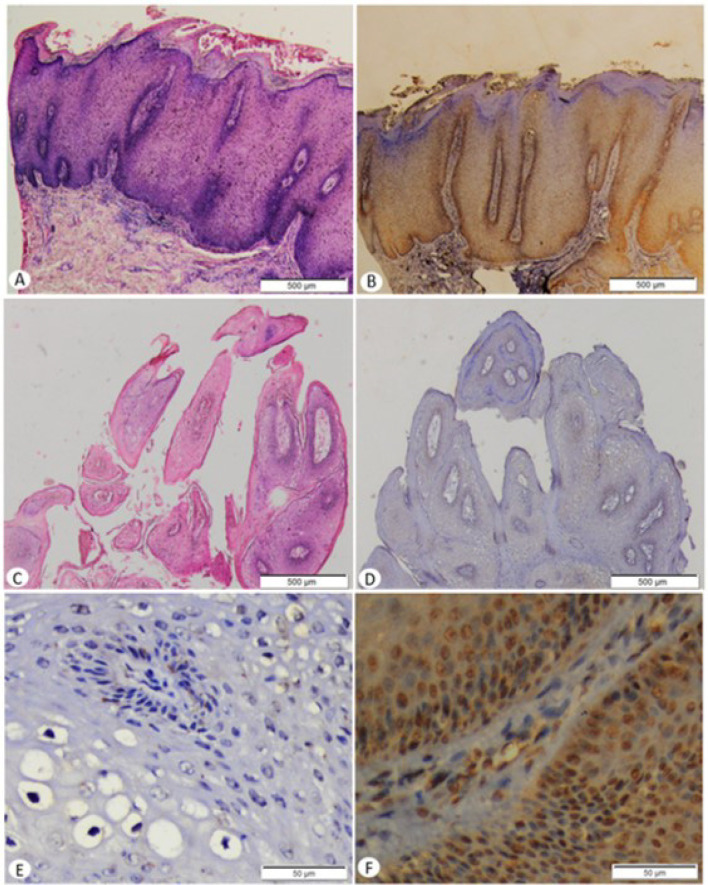
The Histopathological Feature and p16^INK4A^ Expression of Condyloma Acuminata. A, Condyloma acuminata from HR-HPV infected patients, in HE staining (40 x magnification); B, In the same patients, p16^INK4A^ expression showed in diffuse (score 3) (40 x magnification); C, Condyloma acuminata from LR-HPV infected patients, in HE staining (40 x magnification); D, In the same patients, p16INK4A expression showed negative (score 0) (40 x magnification); E, p16^INK4A^ expression in negative (score 0 ) (400 x magnification); F, p16^INK4A^ expression in diffuse (score 3) (400 x magnification)

The expression of p16^INK4A^ in CA lesions showed that nucleus or the combination of nucleus and cytoplasm of cell was stained in brown color, indicating in sporadic, focal, or diffuse (Figure 1). The results showed that 7/33 (21.2%) of the lesions were negative, 13/33 (39.4%) were sporadic, 8/33 (24.2%) were focal, and 5/33 (15.2%) were diffuse. In LR-HPV group, the results showed that negative, sporadic, and focal lesions were 7/19 (36.8%), 9/19 (47.4%), and 3/19 (15.8%), respectively. In HR-HPV group, it was found that that the frequency of sporadic lesions was 4/14 (28.6%), focal was 5/14 (35.7%), and diffuse was 5/14 (35.7%) (Table 3). There was a significant different between LR-HPV and HR-HPV groups in terms of p16^INK4A^ expression in CA lesion (p = 0,000). Correlation between p16^INK4A^ expression and LR-HPV and HR-HPV infections was moderate (r = 0,644, p = 0,000). 

**Table 1:** Characteristics of Patients with Condyloma Acuminata

Characteristics of Patients	N (%)	Single or multiple infection LR/LR HPV N (%)	Multiple infection of LR/HR HPV N (%)
Sex			
Male	12 (36.4)	7 (21.2)	5 (15.1)
Female	21 (63.6)	12 (36.4)	9 (27.3)
Age			
15-24 years	17 (51.5)	10 (30.3)	7 (21.2)
25-34 years	6 (18.2)	3 (9.1)	3 (9.1)
35-44 years	7 (21.2)	4 (12.1)	3 (9.1)
45-54 years	2 (6.1)	1 (3.0)	1 (3.0)
55-64 years	1 (3.0)	1 (3.0)	0
Sexual Partner			
Heterosexuals	30 (90.9)	18 (54.5)	12 (36.4)
Homosexual	1 (3.0)	1 (3.0)	0
Bisexual	2 (6.1)	0	2 (6.1)
Duration of symptom			
< 1 month	5 (15.1)	1 (3.0)	4 (12.1)
1- 3 months	20 (61.6)	14 (42.4)	6 (18.2)
4 - 6 months	6 (18.2)	3 (9.1)	3 (9.1)
> 6 months	2 (6.1)	1 (3.0)	1 (3.0)
History			
First lesion	26 (78.8)	15 (45.4)	11 (33.3)
Recurrent lesion	7 (21.2)	4 (12.1)	3 (9.1)
Lesion on the partner			
Have lesion on the partner	4 (12.1)	2 (6.1)	2 (6.1)
No lesions on the partner	29 (87.9)	17 (51.5)	12 (36.4)
Therapy			
TCA	19 (57.6)	11 (33.3)	8 (24.2)
Cautery	14 (42.4)	8 (24.2)	6 (18.2)
Shape			
Papule	28 (84.8)	16 (48.5)	12 (36.4)
Cauli flower	4 (12.1)	2 (6.1)	2 (6.1)
Flat tapped papule	1 (3.0)	1 (3.0)	0
Type			
Multiple	31 (93.9)	18 (54.5)	13 (39.4)
Solitary	2 (6.1)	1 (3.0)	1 (3.0)
Location			
Penile	7 (21.2)	4 (12.1)	3 (9.1)
Anus	4 (12.1)	2 (6.1)	2 (6.1)
Penile and anus	1 (3.0)	1 (3.0)	0
Labia (majora and minora)	16 (48.5)	10	6 (18.2)
Vulva	3 (9.1)	1 (3.0)	2 (6.1)
Introitus vagina	1 (3.0)	0	1 (3.0)
Perineum	1 (3.0)	1 (3.0)	0

**Table 2:** Distribution of HPV in Condyloma Acuminata Lesions

Genotype of HPV	HR or LR HPV genotypes	Frequency N (%)
LR HPV		19 (57,6)
HPV 6	LR	4
HPV 11	LR	12
HPV 6, 11	LR	2
HPV 6,81,87,89	LR	1
HR HPV		14 (42,4)
HPV 6,11,18,51,82	HR	1
HPV 6,42,51,61	HR	1
HPV 11, 18	HR	2
HPV 11,18,45	HR	1
HPV 11, 26	HR	1
HPV 11,67	HR	1
HPV 11, 51, 82	HR	1
HPV 11, 52, 54	HR	1
HPV 11, 52,69,90	HR	1
HPV 11, 68B	HR	1
HPV 11, 82	HR	2
Total		33

**Table 3:** The p16^ INK4A^ Expression on Condyloma Acuminata that Infected by LR or HR-HPV

p16^ INK4A^ expression	HPV genotype		Total	p-value
	LR-HPV N (%)	LR/HR-HPV N (%)	N (%)	
Score 0 (Negative)	7 (36.8)	0 (0)	7 (21.2)	0.000*
Score 1 (Sporadic)	9 (47.4)	4 (28.6)	13 (39.4)	
Score 2 (Focal)	3 (15.8)	5 (35.7)	8 (24.2)	
Score 3 (Diffuse)	0 (0)	5 (35.7)	5 (15.2)	
Total	19 (100)	14 (100)	33 (100)	

*Mann Whitney, Asymptotic Significance (2-tailed) with p = 0,000 (p < 0,05); Correlation is significant at the 0.01 level (2-tailed) with Spearman's rho (r) = 0,644, p = 0,000)

## Discussion

The microscopic features of CA lesions based on haematoxylin eosin (HE) staining are parakeratosis, hyperkeratosis, hypergranulosis, basal cell hyperplasia, and koilocytic (Léonard et al., 2014). In this study, CA lesions were seen as papules, cauli flowers, and flat-tapping papules. The microscopic features found in this study were hyperkeratosis, parakeratosis, papillomatosis, hypergranulosis, and hyperplasia of basal cell, as well as the koilocytes that usually is associated with HPV infection. 

The most common cause of CA is infection by HPV. There are more than 40 genotypes of HPV that can infect the anogenital area that are usually infected by LR-HPV including HPV 6 and HPV 11 in single infection, but most commonly co-infection with LR-HPV or HR-HPV (Léonard et al., 2014; Hasanzadeh et al., 2019). The results of this study showed that all the tissues taken from the patients, who were clinically diagnosed with CA, were positive for LR-HPV or HR-HPV infections, in single infection or multiple infection. Most of the patients were infected by LR-HPV, while 42.4% were infected by LR-HPV and co-infected with HR-HPV. In line with this study, a cross-sectional study in Kuwait on 156 patients with genital warts showed that 102/156 (65.4%) of the patients were infected by LR-HPV and 54/156 (34.6%) of the patients were infected by HR-HPV. About 88.4% of the patients in the aforementioned study had single infection and 11.6% had multiple infections (Al-Awadhi et al., 2019). Another study in Spain showed that the frequency of LR-HPV infection was 63/138 (45.6%) in their patients and 71/138 (41.4%) of anogenital warts patients were infected by HR-HPV (Arroyo et al., 2016). The other study on 66 anogenital warts specimens showed that LR-HPV infected 42/66 (62.1%) which was dominated by HPV 6 (47%), and HPV-11 (13.6%), as well as HPV 18 and HPV 3 (Ozaydin-Yavuz et al., 2019). We discovered that that beside LR-HPV, CA was also co-infected with HR-HPV that could develop to malignant cancer. Therefore, identification of HPV genotype can predict the risk of developing related diseases. In addition, determination of HPV genotype affects treatment management, patients’ follow-up, and prevention strategies 

The findings of this study revealed that 57.6% of CA patients were infected by LR-HPV and the rest were infected by both LR-HPV and HR-HPV infections. The most common HPV genotypes were HPV 11 and HPV 6 for LR-HPV, followed by HPV 18, HPV 51, and HPV 82 for HR-HPV. The incidence of CA and the progression of the disease can be prevented by vaccination. Recently, there are 3 commercial vaccines against HPV infection, namely Gardasil to prevent infections caused by HPV 6, 11, 16, and 18, Cervarix to prevent infections caused by HPV 16, and 18, and Gardasil 9 to prevent infections caused by HPV 6, 11, 61, 18, 31, 33, 45, 52, and 58 (Gupta et al., 2017). A previous study on anogenital benign lesions showed that HPV genotype in CA patients was dominated by HPV 11 and HPV 6 among 13 female patients (Arista et al., 2019) and 12 male patients (Murtiastutik et al., 2019). In pre-cancerous lesion and cancerous lesion of uterine cervix, those were dominated by HPV 16 (62.68%), then followed by HPV 18 (20.9%), HPV 45 (5.97%), HPV 52 (5.97%), and HPV 67 (4.48%) (Mastutik et al., 2018). Vaccination programs is expected to reduce the incidence of these diseases, but there are some genotypes of HPV that cannot be targeted by the current vaccines. Therefore, strategies to prevent the incidence of CA or the progression of malignancy still need to be developed. 

Oncoprotein E6 and E7 of HR-HPV play a role in cancer development. HR-HPV E6 mediates p53 inactivation by binding to the conserved domains of E6AP (E6-linked protein) to form the E6/ E6AP/p53 complex. This complex causes degradation of p53 by ubiquitination mechanism. HR-HPV E7 targets to degrade the retinoblastoma protein (pRB). In normal cells, when cells are prevented from entering the S phase, the pRB binds to the E2F family of transcription factor, so that the cell stops at the checkpoint of G1-S phase, activating cell cycles arrest. pRb, which is phosphorylated by cyclin D1/CDK 4/6 complex, causes E2F to be released and enter the nucleus. The cell enters the S phase which then starts the activation of gene transcription (Munger et al., 2013, Pal and Kudu, 2020). Furthermore, phosphorylated pRB is a p16^INK4A^ feedback mechanism. HPV E7 induces degradation of pRB by an ubiquitin proteosome pathway that causes the loss feedback mechanism of p16^INK4A^ and then leads to accumulation of p16^INK4A^, presenting as overexpression of p16^INK4A^ (Lassen et al., 2009; Faraji et al., 2017). 

The overexpression of p16^INK4A^ in this study was sporadic (39.4%), focal (24.2%), diffuse (15.2%), and negative (21.2%). Another study on p16^INK4A^ expression in 24 CA specimens showed that overexpression of p16^INK4A^ were sporadic in 11/24 of the specimens (45.8%), were focal in 7/24 of the specimens (29.2%), and were negative in 6/24 of the specimens (25%) (Kazlouskaya et al., 2013). The expression of p16^INK4A^ in cervix tended to increase from cervical normal epithelium to invasive cervical cancer (Missaoui et al., 2010; Izadi-Mood et al., 2012) and cervical adenocarcinoma (Mastutik et al., 2021). The expression of p16^INK4A^ in HR-HPV showed that all specimens were positive ranging from sporadic to diffuse. In LR-HPV were sporadic and focal, whereas 36.8% of specimens were not expressed in p16^INK4A^ and none of specimens expressed in diffuse category. Other study on anal lesion with HR HPV infection showed that all specimens expressed p16^INK4A^ ranging from sporadic to diffuse, whereas those infected with LR-HPV were mostly patchy (Leeman et al., 2019). In oropharyngeal squamous cell carcinomas and tonsillar dysplasia that were positive for HPV 16, p16^INK4A^ expression was also diffuse. However, in benign and pre-malignant lesions were positive for HPV 6 and HPV 11 showed variations from negative to strong positive (Mooren et al., 2014). All cervical lesions infected with HR-HPV had a significant, strong, and diffuse p16^INK4A^ expression, whereas those infected with LR-HPV showed mild p16^INK4A^ expression (score 1) (Missaoui et al., 2014).

These findings highlighted that the expression of p16^INK4A^ significantly correlated with HR-HPV infection, in a way that CA lesions infected by HR-HPV had 0.644 times more chances to express p16^INK4A^ compared to CA lesions infected by LR-HPV. As previous studies showed that p16^INK4A^ expression was associated with HR-HPV infection in oropharyngeal squamous (Liu et al., 2015), mucosal squamous cell carcinomas of the head and neck (Antonsson et al., 2015), cervical squamous intraepithelial lesion (Yildiz et al., 2007), and invasive cervical and vaginal carcinomas (Missaoui et al., 2010; Missaoui et al., 2014). HR-HPV is integrated into the host cell genome, whereas LR-HPV prefers extra chromosome as episome, so that the expression of E6 and E7 oncoproteins are within the regulatory framework of E1 and E2 HPV (Boulet et al., 2007). HR-HPVs, such as HPV 18, 33, HPV 51, HPV 58, and HPV 59, were found to be integrated, while HPVs 30, 35, 39, 44, 45, 53, 56, 59, 74 and 82 did not tend to be integrated, but to be in episome form (Nkili-Meyong et al., 2019). In this study, it was found that HR-HPVs 18, 51, and 82. As reported by Nkili-Meyong et al., (2019), HPV 18 was founded to be integrated in 55% of the positive HPV 18 specimen, HPV 51 was integrated in 25% of the positive HPV 51 specimen, and HPV 82 was founded in extra chromosome. This integration was associated with partial or total deletion of E1 and E2 genes, leading to the overexpression of E6 and E7 due to loss of feedback mechanism by E2 protein (Woodman et al., 2007; Nkili-Meyong et al., 2019). In addition, the HR-HPV E7 oncoproteins have higher affinity to bind pRB than LR-HPV E7, increasing the accumulation of p16^INK4A^. Therefore, all specimens infected by HR-HPV in this study were positive for the expression of p16^INK4A^ in sporadic, focal, and diffuse forms, while those infected by LR-HPV had sporadic and focal, but not diffuse expression. 

In conclusion, this study found significant differences between CA specimens infected by LR-HPV (HPV 6, 11, 42, 61, 54, 81, 87, 89, 90) and HR-HPV (HPV 18, 26, 45, 51, 52, 67, 68B, 69, 82) regarding the expression of p16^INK4A^. p16^INK4A^ expression was correlated with HR-HPV infection moderately. IHC is a routine method to perform the diagnostic of histopathological. Therefore, IHC of p16^INK4A^ could be used as biomarker for HR-HPV infection to predict the malignancy development of CA lesions. 

## Author Contribution Statement

Concepts, design, definition of intellectual content: Gondo Mastutik. Literature search: Gondo Mastutik, Afria Arista. Clinical studies: Gondo Mastutik, Dwi Murtiastutik, Trisniartami Setyaningrum. Experimental studies: Gondo Mastutik, Alphania Rahniayu, Afria Arista. Data acquisition: Nila Kurniasari, Alphania Rahniayu, Anny Setijo Rahaju. Data analysis: Gondo Mastutik, Alphania Rahniayu, Anny Setijo Rahaju.Statistical analysis: Gondo Mastutik, Nila Kurniasari, Anny Setijo Rahaju. Manuscript preparation and manuscript editing: Gondo Mastutik, Afria Arista, Anny Setijo Rahaju. Manuscript review: Gondo Mastutik, Alphania Rahniayu, Dwi Murtiastutik, Trisniartami Setyaningrum, Erna Sulistyani.
